# Factor Structure and Internal Consistency of the Alzheimer's Disease Center's Neuropsychological Test Battery in the Uniform Data Set Version 3: The Nigeria Sample

**DOI:** 10.1002/alz.090255

**Published:** 2025-01-09

**Authors:** Valentine A Ucheagwu, Rita Nonyelum Ugokwe‐Joseph, Jesse Ossai, Chiamaka Odilora, Bruno Giordani

**Affiliations:** ^1^ Global Brain Health Institute Trinity College, Dublin 02 Ireland; ^2^ Nnamdi Azikiwe University Awka, Onitsha / Anambra State Nigeria; ^3^ Nnamdi Azikiwe University, Awka, Anambra Nigeria; ^4^ Michigan Alzheimer's Disease Research Center, Ann Arbor, MI USA; ^5^ University of Michigan, Ann Arbor, MI USA

## Abstract

**Background:**

Harmonized neuropsychological battery for research in Alzheimer’s disease and related dementia (ADRD) in sub‐Sahara Africa is very important if we are to understand ADRD phenotype and mechanisms in the region and how they relate and differ from other regions. No study in Nigeria has examined factor structure (construct validity) at both exploratory and confirmatory levels of harmonized neuropsychological battery for research in Nigeria. This study was a move in this direction using already established neuropsychological test battery the Uniform Data Set Neuropsychological Battery version 3 (UDSNB 3.0) of the Alzheimer’s Disease Center United States.

**Method:**

UDSNB 3.0 fully completed by 349 older adults (220 females) between the 65 – 85 years were used to test for construct validity. Exploratory and confirmatory factor analyses as well as Cronbach Alpha internal consistencies were analyzed.

**Result:**

Principal axis factor analysis (PA) method with orthogonal rotation (varimax with Keiser normalization) and eigenvalue greater than 1 was used for exploratory factor analysis (EFA). Four factors were extracted: first – Executive function/language (Phonemic and category fluencies, number span tasks – forward and backward and multilingual naming test) accounted for 17.23% of the variance, second – Memory (Craft Story immediate and recall) accounted for 16.30%, third factor – Visual spatial (Benson Complex Figure Immediate, recall and recognition) accounting 11.59% and fourth factor – Processing speed accounting 5.64%. Confirmatory factor analysis (CFA) did not fit the four factor exploratory model: CMIN/DF = 4.23; CFI= 0.75; RMSEA=0.12. However, a good model fit was established: CMIN/DF= 2.13; CFI=0.94; RMSEA=0.07, when executive function was unbundled into working memory (Number Span – forward and backward) and language (phonemic and category fluencies) and recognition task of the Benson Complex Figure removed from the model. Moderate internal consistencies were found among the subtests.

**Conclusion:**

UDSNB 3.0 showed good model fit on a five factor structure : Working memory, language, memory, visual spatial and processing speed. These domains could be measured in Nigeria older adults using UDSNB 3.0 cognitive tasks.

